# Treatment of facial myiasis in an elderly patient with oral squamous cell carcinoma: Case report

**DOI:** 10.1016/j.ijscr.2020.05.015

**Published:** 2020-05-21

**Authors:** Samara Andreolla Lazaro, Franklin David Gordillo Yépez, João Paulo De Carli, Micheline Sandini Trentin, Letícia Copatti Dogenski, Ferdinando De Conto

**Affiliations:** a Oral and Maxillofacial Surgery, School of Dentistry University of Passo Fundo, Passo Fundo, RS, Brazil; b Departments of Oral Medicine and Prosthodontics, School of Dentistry, University of Passo Fundo, Passo Fundo, RS, Brazil; c Department of Oral Medicine, School of Dentistry, University of Passo Fundo, Passo Fundo, RS, Brazil; d School of Dentistry of University of Passo Fundo, Passo Fundo, RS, Brazil; e Oral and Maxillofacial Surgery, Coordinator of Department of Oral Surgery, School of Dentistry, University of Passo Fundo, Passo Fundo, RS, Brazil

**Keywords:** Myiasis, Surgical treatment, Epidermoid carcinoma, Aging, Case report

## Abstract

Myiasis is caused by the infestation of fly larvae in human tissues and it presents immunodeficiency, poor hygiene, or malignant neoplasias as predisposing chronic diseases.

**Objective:**

To describe a clinical case of myiasis associated with oral squamous cell carcinoma (OSCC) in an elderly patient.

**Case presentation:**

A 60-year-old male, black, smoker, and alcoholic patient with OSCC, who refused initial cancer treatment and sought hospital care with an extensive facial lesion and approximately 150 larvae in the extraoral region. The treatment was given through the administration of 6 mg of Ivermectin associated with the surgical removal of the larvae. Subsequently, palliative chemotherapy began.

**Discussion:**

Myiasis can sometimes be associated with OSCC and it commonly occurs in individuals who live in unhealthy locations and present poor hygiene and low immunity. In addition to medication, the surgical removal of the larvae is important in the treatment of myiasis.

**Conclusion:**

Patient adherence to cancer treatment is essential to avoid the negative evolution of the tumor. The social component linked to the development of myiasis makes it an important public health topic, as it is related directly to the lack of primary care. Surgical and drug treatments are required to cure myiasis.

## Highlights

• Myiasis may develop secondary to malignant neoplasms.
• Surgical treatment associated with administration of Ivermectin is effective in curing myiasis.
• The establishment of a multiprofessional team is fundamental in the treatment of myiasis.

## 1 Introduction

Myiasis is a parasitic infestation of live vertebrate animals by dipterous larvae, which usually occurs due to poor hygiene in bloody wounds, especially in tropical and subtropical countries [1,2]. Its development is related to poor oral hygiene, alcoholism, senility, or suppurative lesions [3], affecting significantly abandoned elderly people [4,5].

Myiasis can be primary when caused by larvae that feed on living tissue (biophages) and secondary when caused by larvae that feed on dead tissue (scavengers) [2,6]. Myiasis can infest different parts of the body such as the skin and urogenital, ophthalmic, nasopharyngeal, intestinal, and oral cavities [4]. Myiasis can also be associated with carcinomas [1,2,[4], [5], [6], [7], [8], [9], [10], [11], [12]].

Squamous cell carcinoma is the most common malignant neoplasia of the head and neck [13]. Its treatment is managed by a combination of surgery, radiotherapy, and chemotherapy, depending on the place, stage and, TNM classification [14,15]. Quality of life is a major concern in the management of patients with squamous cell carcinoma and it involves a psychological approach. Physical findings such as stench, pain, and oozing may result in severe psychological symptoms, worsening the wounds [7].

Some cases of oral myiasis associated with squamous cell carcinoma have been reported [1,2,[4], [5], [6], [7], [8], [9], [10], [11], [12]]. The condition presents symptoms such as pain, drainage, odor, edema, bleeding, and psychosocial problems that need evaluation and treatment. The presence of larvae in the wound leads to extreme distress for patients [8] as it may remind the decomposition of the body [1].

This study aimed to report a case of myiasis associated with oral squamous cell carcinoma (OSCC) in a 60-year-old Brazilian man, as well as to discuss the adequate diagnosis and treatment for the lesion in question. This study was reported following the SCARE criteria [16].

## 2 Presentation of case

**Fig. 1:**
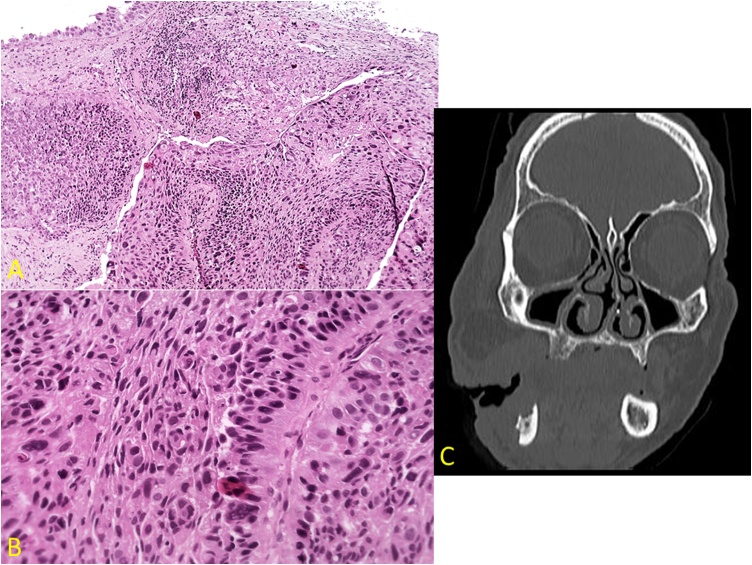
A) The histological section shows a malignant neoplasm of epithelial origin consisting of cells arranged in islands invading the adjacent connective tissue. The stroma of dense connective tissue is permeated by an intense predominantly chronic inflammatory infiltrate (hematoxylin-eosin, 400×); B) The histological section shows malignant cells with cellular/nuclear pleomorphism and hyperchromatism, alteration of the nucleus/cytoplasm ratio, and atypical mitoses (hematoxylin-eosin, 1000×); C) Computed tomography showing extensive facial lesion, communicating with the oral cavity.

Male patient, 60 years old, black, homeless, smoker, and alcoholic, sought hospital care at the Clinics Hospital, complaining of severe pain in the right middle third of the face. The clinical examination revealed diffuse and rigid edema and lymphadenopathy. There was trismus, purulent drainage, and extensive lesion in the alveolar region extending to the oropharynx, with a moriform vegetating aspect and ulcerated areas. The patient was referred to biopsy under general anesthesia and the histopathological report was “squamous cell carcinoma, grade III of the World Health Organization”, with an unfavorable prognosis [Fig. 1]. Subsequently, palliative antineoplastic treatment began, as the lesion was very extensive. However, the patient did not adhere to the treatment and evaded the hospital.

**Fig. 2:**
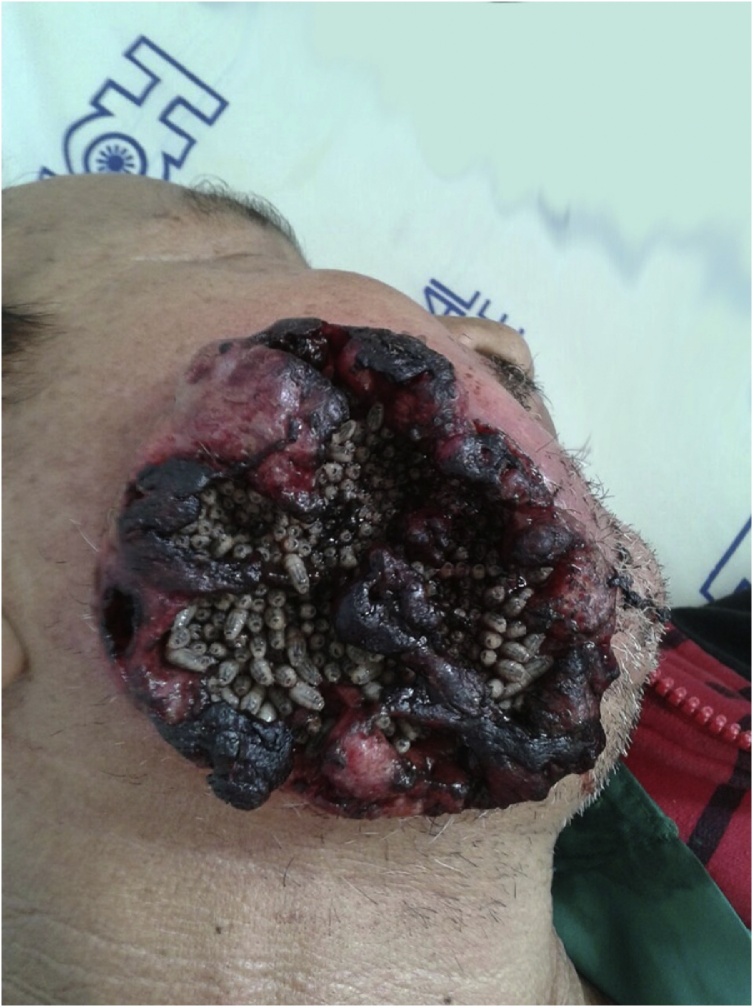
Extensive facial lesion with presence of multiple larvae.

After two months, the patient returned and palliative chemotherapy restarted with 2500 mg of Fauldfluor +50 mg of Cisplatin, with 21-day cycles. However, patient evasion was recorded again. Three months after the beginning of chemotherapy, the patient returned complaining of severe pain in the middle third of the face and dysphagia. He verbalized with difficulty, had edema, erythema, and extensive lesion comprising the middle and lower third region of the right side of the face, with necrotic and elevated edges, loss of skin substance, communication with the oral cavity, and approximately 150 larvae in extraoral region [Fig. 2].

Oroscopy revealed an ulcerated vegetating tumor locally infiltrated advancing in the oral cavity with extension to the oropharynx, tonsillar, and soft palate, with jaw invasion and extraoral extension in the right jugal region with cutaneous involvement (T4), and levels I and II cervical lymph node enlargement of up to 2 cm in diameter (N2). The complete surgical removal was not possible so palliative cancer treatment was indicated. The tomography of the cervical region suggested lesion in the oral cavity with complete erosion of the mandibular body and extension to the submandibular region, extending to the tonsillar pillar and right vallecula, measuring about 7.2 × 7.7 × 7.6 cm and lymph node enlargement in the Ib, II and III chains on the right side, measuring up to 2.0 × 1.6 cm.

**Fig. 3:**
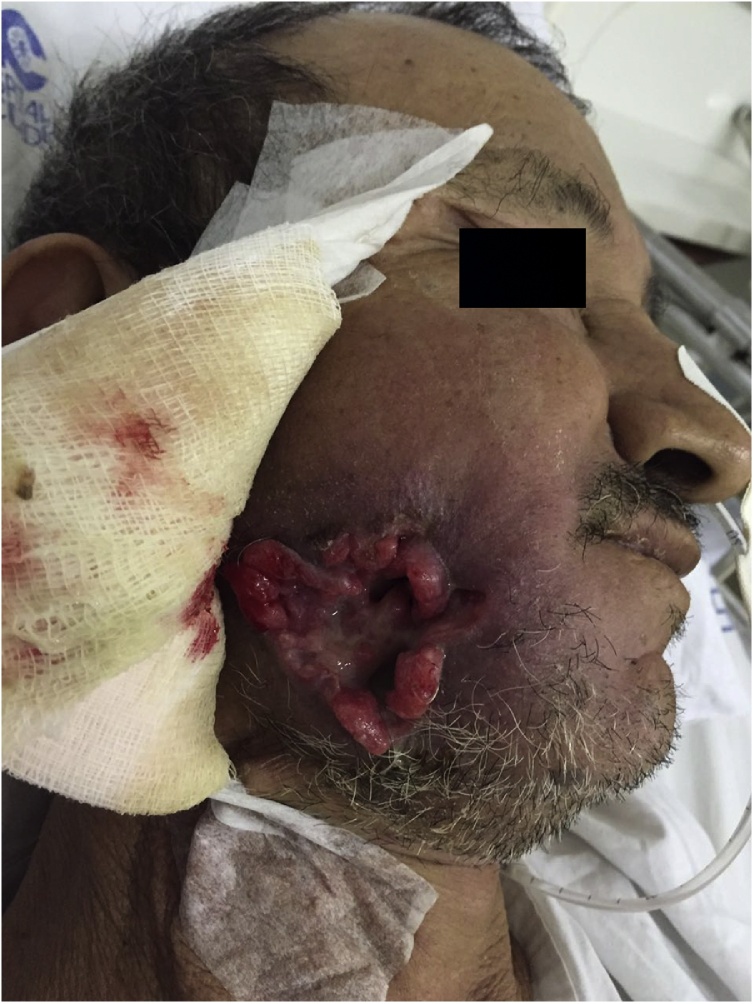
Clinical appearance after complete larvae removal.

**Fig. 4:**
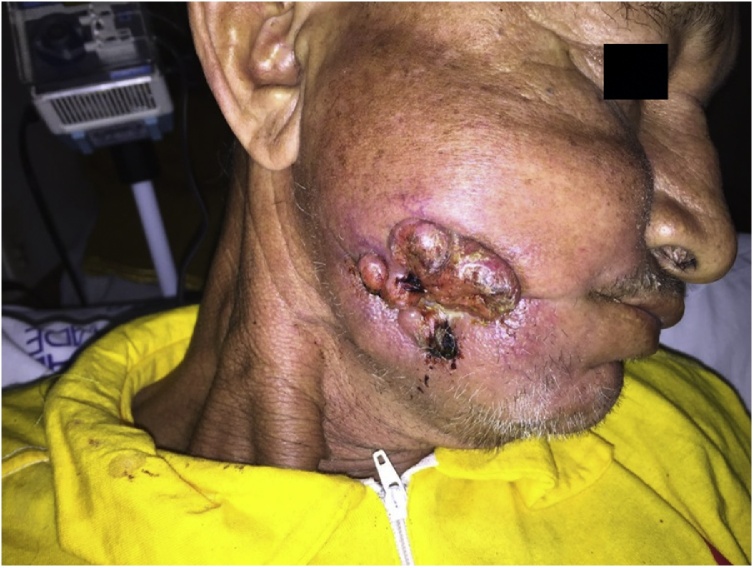
Appearance during antineoplastic treatment.

**Fig. 5:**
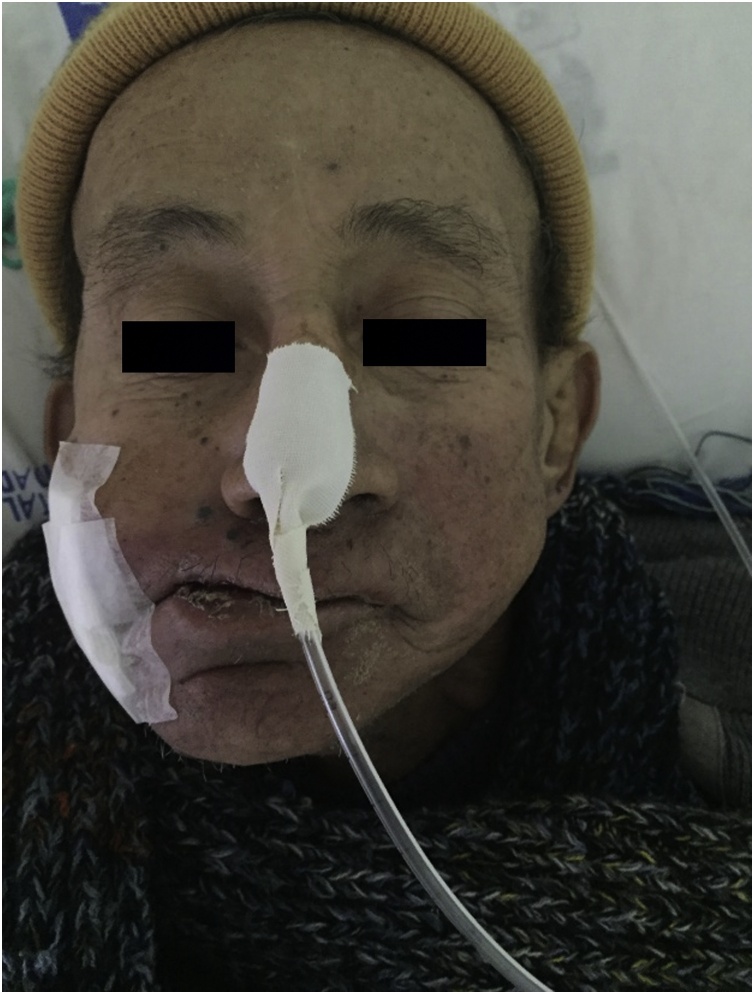
In palliative care, two weeks before death.

Treatment was performed with the oral administration of 6 mg of Ivermectin for three days. The larvae were removed at three stages: superficial skin larvae were removed in the emergency department, the other 17 larvae located in deeper regions were removed in the operating room, and eight larvae were removed at the hospital bed. After complete removal [Fig. 3], the patient started palliative chemotherapy and he was referred to radiotherapy [Fig. 4] and follow-up with a social worker and a psychology team. After four months, the patient died [Fig. 5].

## 3 Discussion

The increase of the elderly population is accompanied by a higher incidence of diseases [17]. The oral health of the elderly is often neglected due to the lack of preventive care. Most elderly people tend to dismiss oral health services because they believe they no longer need or deserve this type of care, which aggravates their health condition and self-perception [18].

The association between low socioeconomic conditions and head and neck cancer is also described. Patients with larvae-infested oral malignancy are often negligent with advanced cases of oral squamous cell carcinoma (OSCC), poor socioeconomic status, and poor oral hygiene [1]. The profile (homeless) of the patient described in this study includes him directly in the risk group for the development of such disease, considering that unhealthy conditions, advanced age, medical comorbidities, poor access to health services, and lack of knowledge are predisposing factors to myiasis [1].

Extensive oral myiasis infestation associated with OSCC is reported in the literature, but there are few expressive articles [1,2,4,5,[8], [9], [10], [11], [12]], which are restricted to isolated cases or case series studies. Thus, it may be said that the frequency of the association between myiasis and OSCC is small or underreported.

A recent systematic review [6] performed a literature search on PubMed, Medline, and Cochrane databases on November 1, 2018 for all the articles focusing on oral myiasis in patients with OSCC. According to the authors, nine cases of oral myiasis in association with OSCC have been reported to date. Out of these nine cases, five have been reported from India and four from Brazil. Girardi and Scrofernecker [1] described a case of a Brazilian patient from upstate Rio Grande do Sul, with OSCC associated with myiasis. These results are consistent with the statement by Pessoa and Galvão [9], that myiasis is mainly found in tropical countries, agreeing with the characteristics of the patient described in the present study, considering he was a Brazilian man from upstate Rio Grande do Sul [Table 1].

**Table 1:** Clinical and epidemiological characteristics of some relevant cases reported that showed association between myiasis and OSCC.

Study	Country of origin	Number of cases	Sex	Location of the tumor	Age (absolute/average)	Treatment	Follow-up
Carvalho et al. [10]	Brazil	01	M	Buccal mucosa	80	Larvae removal and debridement of necrotic tissues	Death due to complications of the larval infestation after 3 months
Gabriel et al. [11]	Brazil	01	F	Lower jaw	72	Larvae removal and debridement of necrotic tissues	Patient was referred to cancer hospital and did not return for follow-up
Pessoa and Galvão [9]	Brazil	01	M	Lower jaw	44	Larvae removal and cleaning with 0.12 % chlorhexidine	Death by cancer progression after 2 months
Dharshiyani et al. [12]	India	01	M	Lower jaw	75	Larvae removal and systemic antibiotics + oral Ivermectin	Patient referred to cancer hospital and did not return for follow-up
Biradar et al. [4]	India	02	M	Oral mucosa	525	Larvae removal and systemic antibiotics (amoxicillin/ clavulanic acid/ metronidazole)	Patients did not return for further treatment and follow-up
Girardi and Scrofernecker [1]	Brazil	01	M	Upper jaw	67	Larvae removal and oral Ivermectin	Death by cancer progression after 1 year

Myiasis presents several therapeutic options. The simplest one is the mechanical removal of larvae under local anesthesia or surgical debridement. The literature also quite successfully reports the use of Ivermectin, a broad-spectrum semi-synthetic macrolide antibiotic [8]. It activates the release of γ-aminobutyric acid, which induces larval death and spontaneous elimination [4,19]. In this study, Ivermectin administered for three days associated with the surgical removal of larvae was effective in the treatment of myiasis.

One of the great challenges of the multi-professional team working in the care of cancer patients is to obtain patient adherence to treatment. The importance of promoting strategies to establish bonds that provide space for educational and psychosocial intervention is emphasized so that patients develop the necessary motivation [20]. In the case reported, it is clear the patient's refusal for the treatment was attributed to factors such as misinformation and fear. The Public Health System of Brazil (*Sistema Único de Saúde*, SUS) offered full access to cancer treatment and yet the patient evaded the hospital on two occasions.

## 4 Conclusion

Professionals should encourage the maintenance of good hygiene to their patients. The need to establish a multi-professional team is essential, considering myiasis in humans occurs in individuals with an unbalanced self-care. The surgical treatment associated with the administration of Ivermectin was effective in curing myiasis, although the patient's non-adherence to cancer treatment anticipated his death.

## Declaration of Competing Interest

None of the authors has any conflict of interest.

## Sources of funding

The authors state that the present study had no sponsor or source of funding.

## Ethical approval

Because this is a case report, the present study was not appreciated by a research ethics committee. However, it follows as an attached file a patient's fully informed written consent for publication of the reported clinical case.

## Consent

Written informed consent was obtained from the patient for publication of this case report and accompanying images. A copy of the written consent is available for review by the Editor-in-Chief of this journal upon request.

## Authors contribution

Samara Andreolla Lazaro – Execution of the surgical step; acquisition of data.

Franklin David Gordillo Yépez – Execution of the surgical step; acquisition of data.

João Paulo De Carli – Writing work, discussion and final approval; conception and design of the study.

Micheline Sandini Trentin – Writing work, discussion and final approval; conception and design of the study.

Letícia Copatti Dogenski – Literature review, translation and spelling revision; conception and design of the study.

Ferdinando De Conto – Execution of the surgical step; acquisition of data.

## Registration of research studies

The present study is not a research involving humans, but a clinical case report, whose patient authorized the publication by means of a free and informed consent term.

## Guarantor

João Paulo De Carli.

## Provenance and peer review

Not commissioned, externally peer-reviewed.
